# The High-Pressure Response of 9,9′-Spirobifluorene Studied by Raman Spectroscopy

**DOI:** 10.3390/molecules30030638

**Published:** 2025-01-31

**Authors:** Maria-Tereza Siavou, Konstantina Siapaka, Olga Karabinaki, Dimitrios Christofilos, John Arvanitidis

**Affiliations:** 1 Physics Department, Aristotle University of Thessaloniki, 54124 Thessaloniki, Greece; msiavou@physics.auth.gr (M.-T.S.); ksiapaka@physics.auth.gr (K.S.); 2 School of Chemical Engineering & Laboratory of Physics, Faculty of Engineering, Aristotle University of Thessaloniki, 54124 Thessaloniki, Greece; okarabi@auth.gr (O.K.); christop@cheng.auth.gr (D.C.)

**Keywords:** polycyclic aromatic hydrocarbon (PAH), 9,9′-spirobifluorene, Raman spectroscopy, high pressure, structural transition

## Abstract

The pressure response of crystalline 9,9′-spirobifluorene up to 8 GPa was studied by means of Raman spectroscopy using a diamond anvil cell as a pressure chamber. With increasing pressure, the observed Raman peaks shifted to higher frequencies, reflecting the bond hardening upon volume reduction, which was much more pronounced for the initially weaker intermolecular interactions than for the stronger intramolecular covalent bonds. The significant changes in the Raman spectrum and the pressure evolution of the frequencies at ~1.3 GPa for both the intermolecular and the intramolecular Raman peaks signaled a pressure-induced structural and molecular conformation transition with a little hysteretic behavior (~0.5 GPa) upon pressure release. For *P* > 4 GPa, the reversible decrease of the pressure coefficients of the majority of the intermolecular and some intramolecular peak frequencies indicated another structural modification of the studied molecular crystal. A value of ~9 GPa for the bulk modulus of the system at zero pressure was estimated from the logarithmic pressure coefficients of the frequencies of the intermolecular modes in the low-pressure phase. These coefficients were reduced by ~6 times at 4.2 GPa, indicating that the considerable stiffening of the material in the high-pressure phase emanated from the selective strengthening of the intermolecular interactions.

## 1. Introduction

Molecular crystals, which consist of molecules held together by weak intermolecular interactions—such as van der Waals and hydrogen bonding—are highly significant in the fields of material science, chemistry and life science. Among the organic molecules, polycyclic aromatic hydrocarbons (PAHs) are a diverse group of molecular structures made up of two or more interconnected aromatic rings. Apart from their intrinsic toxicity [1,2,3,4,5], these compounds have garnered considerable interest because of their unique physical and chemical properties due to the delocalization of π-electrons within their aromatic rings, strongly influenced by the exact molecular structure [1,4,6,7,8,9,10,11,12]. Therefore, PAHs and their derivatives have diverse applications in pharmaceuticals, plastics, dyes, liquid crystals and optoelectronics [7,8,9,10,11]. The formation of spiro compounds is a very promising way to improve the morphological stability—while retaining the functionality—of PAH molecules with a relatively low molecular weight, which is vital for optoelectronic applications [13,14]. Spiro compounds consist of two mutually perpendicular π-systems connected through a common tetracoordinated atom [15]. The perpendicular arrangement of the two parent molecular units leads to the high steric demand of the resulting rigid structure. This structural feature efficiently diminishes molecular interactions between the π-systems, which leads to the higher solubility of the spiro-linked compounds. The spiro-linking also considerably suppresses the excimer formation, frequently observed in the solid state, stabilizing the emission properties [16].

The 9,9′-spirobifluorene (SBF, C_25_H_16_) molecule consists of two fluorene-like subunits—each composed of two aromatic hexagonal rings connected by a non-aromatic pentagonal ring—in a nearly perpendicular configuration [17]. It exhibits the *D*_2*d*_ point group symmetry and thus presents an intriguing structural motif that has been applied in molecular recognition and catalysis [18,19] and integrated into materials with unusual optoelectronic properties [20]. SBF crystallizes in the monoclinic structure (space group: *P*2_1_/*c*, lattice parameters: *a* = 10.173 Å, *b* = 10.241 Å, *c* = 16.722 Å, *β* = 96.52°, Z = 4), where the individual rings remain planar but the biphenyl groups of the molecule deviate significantly from planarity due to crystal packing [21]. Note that the formation of another, possibly metastable SBF polymorph has also been reported, having the same space group but larger lattice parameters: *a* = 18.2491 Å, *b* = 11.1522 Å, *c* = 18.6918 Å, *β* = 117.907° and Z = 8 [22]. This difference arises from the fact that the metastable polymorph contains two SBF molecules in the asymmetric unit instead of one in the case of its stable counterpart, owing to the formation of centrosymmetric SBF dimers through C-H···π(arene) hydrogen bonding [22].

Because of the coexistence of weak intermolecular interactions and strong intramolecular covalent bonds in molecular crystals, the application of pressure acts selectively, reducing much more pronouncedly the intermolecular distances. This is usually followed by changes in the relative orientations of the constituent molecules and/or structural transitions—even at moderate pressures—without altering their chemical structure. Raman spectroscopy, which probes in a facile way both intermolecular (external) and intramolecular (internal) vibrations, is usually the method of choice to investigate the response of molecular crystals and the different evolution of the inter- and the intramolecular modes with pressure [23,24,25]. The parent benzene and several PAHs that are comprised of 2–4 hexagonal aromatic rings (naphthalene, biphenyl, fluorene, anthracene, phenanthrene, tetracene, chrysene, pyrene and triphenylene) have been studied by means of Raman spectroscopy under high pressure, and subtle structural transitions at moderate pressures (below 8 GPa) were reported [26,27,28,29,30,31,32,33,34,35,36,37,38,39]. These transitions are usually sluggish and reversible and are associated with symmetry changes, molecular reorientations and stacking rearrangements, charge transfer processes, or changes in the π-electron density [34,37].

Here, we report, for the first time, the structural stability and pressure-induced phase transitions of SBF crystals, adding to the existing knowledge of the physical properties of PAH systems. Further, an estimation of the bulk modulus of SBF is provided, which is not available in the literature. Such information is valuable for the potential use of PAHs in the various applications aforementioned, as well as for understanding the pressure response of molecular crystals in general. More specifically, high-pressure Raman measurements of SBF crystals were conducted up to 8 GPa using a diamond anvil cell (DAC) for pressure application and Daphne 7474 as a pressure transmitting medium (PTM). Significant alterations in the Raman spectrum profile and the pressure evolution of the Raman peak frequencies at ~1.3 GPa were observed, which were attributed to a pressure-induced structural and molecular conformation transition of SBF with small hysteresis upon pressure release. Above 4 GPa, the reversible changes in the pressure coefficients of the majority of the intermolecular and some intramolecular peak frequencies in the low (<300 cm^−1^) and 480–900 cm^−1^ frequency regions indicated either a second structural transition of the system or the completion of the first one at ~1.3 GPa.

## 2. Results and Discussion

### 2.1. Raman Spectrum of SBF at Ambient Pressure

**Figure 1:**
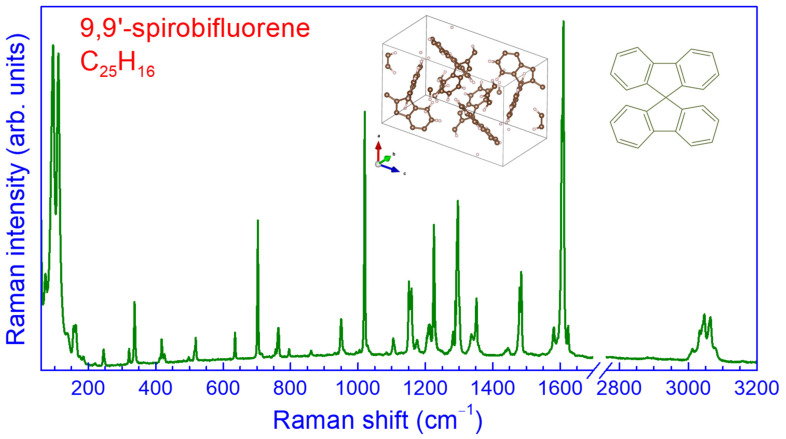
Typical Raman spectrum of 9,9′-spirobifluorene (SBF) at ambient pressure, excited at 632.8 nm. Insets show a schematic representation of the SBF molecule and the unit cell of the crystal structure [21,42].

The Raman spectrum of the crystalline SBF at ambient conditions excited with λ_exc_ = 632.8 nm is illustrated in Figure 1. Because of the complexity of the studied system and the large number of atoms included in its molecule and monoclinic unit cell, the spectrum was very rich in terms of the number of observed peaks and could be divided into three main frequency regions. In the frequency region below 250 cm^−1^, intermolecular (external) vibrational modes and intramolecular (internal) torsional and skeleton deformation modes are expected due to the smaller values of the corresponding force constants and the heavier masses involved. The majority of the intramolecular modes exhibited frequencies above 250 cm^−1^ owing to the strong covalent bonding and the relative motion of lighter masses. In the frequency region 300–1600 cm^−1^, the intramolecular vibrations included C–H and CCC bending and ring deformations in the two fluorene-like subunits, while C–H stretching vibrations are expected in the frequency region 2900–3250 cm^−1^. In this high-frequency region, higher-order (overtones and combinational) vibrational modes with relatively low intensities are also expected, particularly in the range of 3100–3250 cm^−1^. Contrary to the case of the fluorene molecule, the absence of the CH_2_ methylene group in SBF—due to the formation of the spiro-linking—caused the disappearance of the corresponding stretching vibrations in the frequency region 2900–3000 cm^−1^ of the Raman spectrum [37,40,41]. Thus, the C-H stretching vibrations in SBF were limited in the range of 3000–3100 cm^−1^.

The frequency positions of the various Raman peaks appearing in Figure 1 and their relative intensities were in fair agreement with those reported earlier for SBF crystals by Boo et al. using λ_exc_ = 514.5 nm [43]. Boo et al. also assigned their experimentally observed Raman peaks to specific intramolecular vibrational modes by means of ab initio Hartree–Fock (HF) and Becke 3 Lee–Yang–Parr (B3LYP) density functional theory (DFT) calculations using the 4–31 G basis set. They further confirmed their assignment for some of the peaks by solution Raman measurements with the depolarization method of SBF in CCl_4_ [43]. For the isolated SBF molecule with 41 atoms and the *D*_2*d*_ point group symmetry, 88 fundamentals are expected, with symmetries 20A_1_ + 9A_2_ + 10B_1_ + 20B_2_ + 29E. From these, the A_2_ modes are inactive in both Raman and infrared (IR) spectroscopy, while the A_1_ and B_1_ modes are active only in Raman scattering. On the other hand, the B_2_ and E modes—the latter were doubly degenerate modes because of the presence of two identical fluorene-like subunits in the SBF molecule—are active in both Raman and IR spectroscopy [43].

In this work, we adopted the same assignment for the various Raman peaks by comparing the extrapolated frequencies to zero pressure from the low-pressure ω vs. *P* data with those experimentally obtained at ambient conditions by Boo et al. (Appendix A, Table A1). Several additional peaks appearing in the Raman spectrum of crystalline SBF were also tentatively assigned to intramolecular or intermolecular (external) vibrations (low-frequency region). This was accomplished by taking into account their similar ambient pressure frequencies to those obtained from the DFT calculations by Boo et al. for the SBF molecule in the *D*_2*d*_ point group symmetry [43] and assuming a possible crystal field and molecular deformation [21] splitting of the degenerate E modes (shown in italics in Table A1). Some weak Raman peaks with relatively high frequencies could not be assigned to specific fundamentals of the SBF molecule and may have originated, as also mentioned above, from second-order Raman scattering. Noticeably, the similarity of some Raman peak frequencies of SBF with those of fluorene monomer and other fluorene-containing spiro compounds (dicarbomethoxy and dimethyl-substituted spiro-cyclopropane-1,9′-fluorene as well as bis(2,2′-biphenylene)silane and bis(2,2′-biphenylene)germane), was remarkable, being indicative of their common origin [40,41,43,44,45].

### 2.2. Pressure Dependence of the Raman Spectrum in the Low- and High-Frequency Regions

In this subsection, the results of the high-pressure Raman study of SBF in the low- (ω < 300 cm^−1^) and high-frequency (3000–3250 cm^−1^) regions of the spectrum are summarized and discussed. As mentioned in the previous subsection, the first region contained the intermolecular and intramolecular torsional and skeleton deformation modes, while the second included C–H stretching vibrations. The character of the former modes and the peripheral positions of the hydrogen atoms, associated with the latter vibrations, rendered these modes more sensitive—compared to the intermediate frequency intramolecular modes—to volume contraction and the possible occurrence of any pressure-induced structural and/or molecular conformation modifications [37].

**Figure 2:**
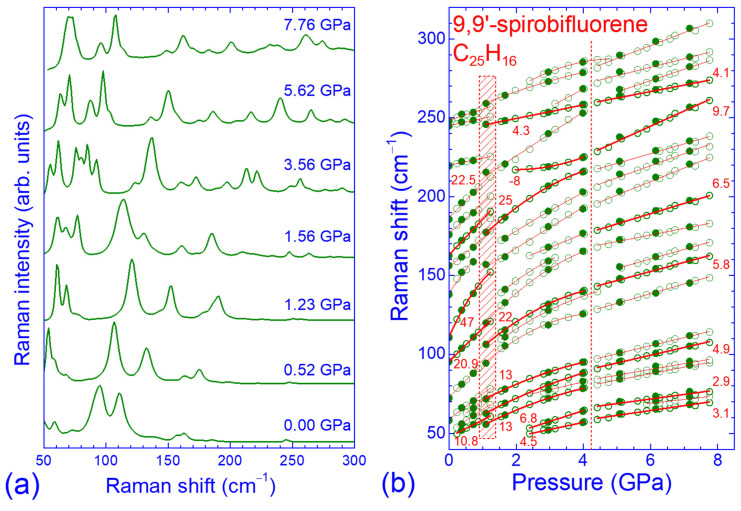
(**a**) Raman spectra of SBF in the frequency region of the intermolecular vibrations recorded at various pressures. (**b**) Pressure dependence of the corresponding Raman peaks. Open (solid) symbols denote data obtained upon pressure increase (decrease). Solid lines through the experimental data are their linear or parabolic least-square fits, while numbers refer to the linear pressure coefficients of the well-resolved Raman peak frequencies (thicker symbols and lines). The shaded area and the vertical dashed line denote pressure ranges where changes in the Raman peak frequencies and their pressure coefficients occurred.

Representative Raman spectra of SBF at various pressures in the low-frequency region are illustrated in Figure 2a. With increasing pressure, all Raman peaks shifted to higher frequencies, reflecting the bond hardening upon volume contraction. Moreover, considerable relative intensity changes of the various peaks were also observed in the whole pressure range investigated (0–8 GPa). Bond hardening with increasing pressure forced additional Raman peaks, initially located below 50 cm^−1^, to gradually enter the spectral window depicted in the figure (peaks ω_1_, ω_3_, ω_4_, ω_6_ and ω_7_ in Table A1). Note that the low-frequency edge of this spectral region was limited by the edge filter cut-off, which was used in the spectrometer to eliminate the strong, elastically scattered light (Section 3). Apart from these observations, abrupt changes regarding the appearance of the Raman peaks and their frequency shifts with pressure were apparent when going from 1.23 to 1.56 GPa, signaling the occurrence of a structural phase transition.

The pressure dependence of the frequencies of the various Raman peaks in the low-frequency region is illustrated in Figure 2b. For each peak, the ω vs. *P* data upon pressure increase (open circles) and decrease (solid circles) were fitted by linear (ω = ω_0_ + *b*_1_*P*) or parabolic (ω = ω_0_ + *b*_1_*P* + *b*_2_*P*^2^) functions in three different pressure ranges (*P* < 1.3 GPa, 1.1 < *P* < 4.1 GPa and *P* > 4.2 GPa). The linear pressure coefficients (*b*_1_) of the stronger peaks (thicker circles and lines) are also shown in the figure. The frequencies ω_0_ obtained from these fits and the corresponding pressure coefficients *b*_1_ and *b*_2_ for all the observed Raman peaks are summarized in Table A1 (Appendix A). As can be inferred from Figure 2b, the pressure dependencies of the frequencies for both intramolecular and intermolecular modes were smooth and linear up to ~1.3 GPa, with the exception of the external ω_15_ mode, which showed a strong parabolic dependence. The Raman peaks with frequencies below 200 cm^−1^ exhibited relatively large pressure coefficients, compatible with the small force constants involved [24]. However, abrupt changes and discontinuities occurred above this pressure value. More specifically, the Raman peaks attributed to intermolecular vibrations ω_7_, ω_17_ and ω_20_ disappeared, whereas new Raman peaks (ω_5_, ω_10_, ω_13_, ω_14_, ω_24_ and ω_26_) progressively appeared in the spectrum as pressure increased from 1.5 to 3 GPa. In addition, as the pressure increased from 1.23 to 1.56 GPa, the frequencies of several Raman peaks attributed to intermolecular (ω_8_ and ω_19_) and intramolecular (ω_4_, ω_6_, ω_11_, ω_18_, ω_19_, ω_22_ and ω_23_) modes either remained constant or softened by 1–8 cm^−1^. This behavior indicated a weakening of the corresponding intermolecular and intramolecular interactions within this pressure range, probably caused by the abrupt anisotropic shift of the molecular units from their initial positions in the unit cell. The pressure dependencies (pressure coefficients *b*_1_ and *b*_2_) of the various peak frequencies were also different below and above 1.3 GPa. All these pressure-induced peculiarities were compatible with the aforementioned evolution of the overall Raman spectrum profile when going from 1.23 to 1.56 GPa (Figure 2a) and supported the hypothesis of a pressure-induced structural and molecular conformation transition for *P* > 1.3 GPa. This transition showed some hysteresis (~0.5 GPa) upon pressure release (solid symbols in Figure 2b); the high-pressure phase survived down to ~1.1 GPa and the low-pressure phase was recovered at ~0.7 GPa, suggesting the first-order nature of the observed phase transition. Since there was no significant change in the number of the intermolecular modes appearing in the Raman spectrum in the low- and high-pressure phases, the crystal structure most probably remained monoclinic.

In the pressure region 1.3–4 GPa, the pressure dependencies of the frequencies of the low-frequency Raman peaks were mainly sublinear (negative quadratic pressure coefficients, *b*_2_), reflecting the gradual strengthening of the initially weak intermolecular interactions upon volume contraction and the stiffening of SBF [25,37]. Noticeably, the Raman peak ω_22_, possibly originating from an intramolecular mode associated with ring deformation, showed peculiar behavior upon compression. Its frequency remained nearly constant within the pressure range of 2–3 GPa, whereas it increased quasi-linearly for *P* > 3 GPa, resulting in an overall superlinear pressure response (positive pressure coefficient *b*_2_) in the pressure range under consideration (1.3–4 GPa). For pressures higher than 4 GPa, all the observed Raman peaks exhibited linear ω vs. *P* dependencies with reduced pressure coefficients *b*_1_ (Figure 2b). The decrease of the pressure coefficients indicated the considerable stiffening of the studied molecular crystals at elevated pressures. We recall that similar behavior has also been observed in the case of the parent orthorhombic fluorene for *P* > 6 GPa [37]. High-pressure X-ray diffraction (XRD) measurements have shown that fluorene undergoes a pressure-induced isostructural phase transition for *P* > 3 GPa, caused by the molecular rearrangement from the ambient and low-pressure herringbone pattern to π-stacking, resulting in abrupt changes in the lattice parameters (increase in *a* and *b* and decrease in the *c* lattice constant). The low- and high-pressure phases co-existed up to ~5 GPa, where crystalline fluorene was completely transformed to the stiffer high-pressure phase [46]. This structural transition was also apparent in the pressure evolution of the Raman peak frequencies since two distinct reversible changes in the linear pressure coefficients—mainly to lower values—were observed at 2.5 and 6 GPa, but without any spectroscopic evidence for phase coexistence in the intermediate pressure region [37]. In the case of SBF, the absence of high-pressure XRD data in the literature prevented us from conclusively judging whether two different structural phase transitions occurred at ~1.3 and ~4.2 GPa or whether a single one—sluggish, with phase coexistence—took place in the pressure range of 1.3–4.2 GPa, possibly characterized by the reorientation and different stacking of the SBF molecules.

**Figure 3:**
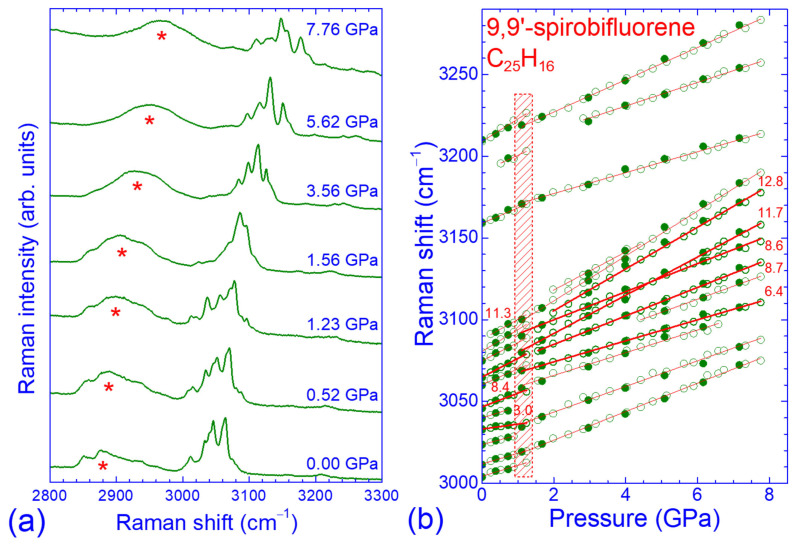
(**a**) Raman spectra of SBF in the frequency region of the C-H stretching vibrations recorded at various pressures. Asterisks mark the C-H stretching band of the pressure-transmitting medium. (**b**) Pressure dependence of the corresponding Raman peaks. Open (solid) symbols denote data obtained upon pressure increase (decrease). Solid lines through the experimental data are their linear least-square fits, while numbers refer to the pressure coefficients of the well-resolved Raman peak frequencies (thicker symbols and lines). The shaded area denotes the pressure range where changes in the Raman peak frequencies and their pressure coefficients occurred.

Representative high-pressure Raman spectra of SBF in the high-frequency intramolecular mode region, including C–H stretching vibrations and higher-order modes as well as the pressure evolution of the corresponding Raman peak frequencies, are illustrated in Figure 3. Similarly to the low-frequency region, all Raman peaks shifted to higher frequencies with increasing pressure and the concomitant volume contraction. The pressure dependencies of the peak frequencies were quasi-linear up to ~1.3 GPa, where the pressure-induced phase transition took place and the overall spectrum profile changed significantly when going from 1.23 to 1.56 GPa. The ω vs. *P* dependencies for all the observed peaks were also quasi-linear up to the maximum pressure attained in our experiments. Nevertheless, the pressure coefficients of the peak frequencies above 1.3 GPa were different from those for lower pressures; the coefficients at high pressures increased for the first-order Raman peaks ω_97_, ω_103_ and ω_106_, attributed to C–H stretching vibrations, and the second-order Raman peak ω_95_, but decreased for the first-order Raman peaks ω_99_, ω_100_, ω_102_ and ω_104_, attributed to C–H stretching vibrations, and the high-frequency second-order Raman peaks ω_108_–ω_110_. The ω vs. *P* data for the high-frequency intramolecular modes exhibited the same hysteretic behavior upon pressure release to that of the intermolecular and low-frequency intramolecular modes, as the low-pressure phase of SBF was recovered at ~0.7 GPa.

### 2.3. Pressure Dependence of the Raman Spectrum in the Intermediate-Frequency Region

**Figure 4:**
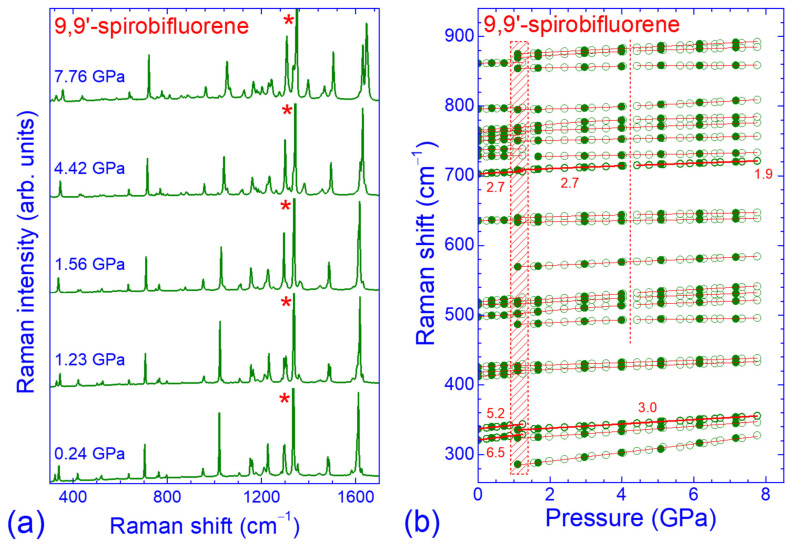
(**a**) Raman spectra of SBF in the frequency region of 300–1700 cm^−1^ recorded at various pressures. Asterisks mark the strong phonon peak of the diamond anvil. (**b**) Pressure dependence of the frequencies of the intramolecular modes in the frequency region of 280–920 cm^−1^. Open (solid) symbols denote data obtained upon pressure increase (decrease). Solid lines through the experimental data are their linear or parabolic least-square fits, while numbers refer to the linear pressure coefficients of the well-resolved Raman peak frequencies (thicker symbols and lines). The shaded area and the vertical dashed line denote pressure ranges where changes in the Raman peak frequencies and their pressure coefficients occurred.

Raman spectra of SBF at selected pressures in the intermediate-frequency region of the intramolecular modes (300–1700 cm^−1^) are illustrated in Figure 4a.

**Figure 5:**
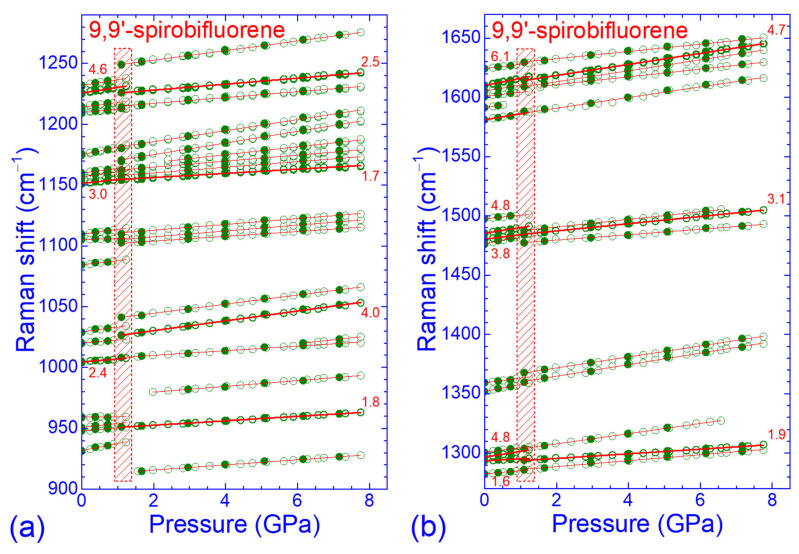
Pressure dependence of the frequencies of the intramolecular modes of SBF in the frequency regions (**a**) 900–1290 cm^−1^ and (**b**) 1270–1670 cm^−1^. Open (solid) symbols correspond to data obtained upon pressure increase (decrease), lines through the experimental data are their least-square fits and numbers refer to the linear pressure coefficients of the well-resolved Raman peak frequencies (thicker symbols and lines). The shaded area denotes the pressure range where changes in the Raman peak frequencies and their pressure coefficients occurred.

The Raman peaks in this frequency region also shifted to higher frequencies upon compression but with smaller rates compared to the Raman peaks in the low- and high-frequency regions. Moreover, the changes in the overall spectral profile were also much less pronounced and compatible with their internal character. The pressure dependencies of the frequencies of the observed Raman peaks in the intermediate-frequency region are shown in Figure 4b and Figure 5. Changes in the pressure evolution of the peak frequencies as well as the appearance or disappearance of some Raman peaks were also observed at ~1.3 GPa, with some hysteresis upon pressure release. The majority of the Raman peaks exhibited quasi-linear ω vs. *P* dependencies in the pressure ranges of 0–1.3 GPa and 1.1–7.8 GPa, with generally smaller pressure coefficients in the high-pressure phase. However, there were several Raman peaks in the frequency region of 480–900 cm^−1^, attributed to in- and out-of-plane C–H and CCC bending (Table A1) that had parabolic ω-*P* dependencies for 1.1 < *P* < 4.1 GPa and/or underwent changes in the linear pressure coefficients of their frequencies above 4.2 GPa.

### 2.4. Force Hierarchy and Elastic Properties of SBF

The mode Grüneisen parameter γ_i_ = *B*_0_·∂(lnω_i_)/∂*P* (where *B*_0_ is the bulk modulus and ω_i_ is the frequency of the *i*th mode) is related to the pressure-induced volume contraction and the concomitant modifications of the force constants of the bonds involved in the vibrational mode. In the Grüneisen approximation, all the γ_i_ parameters of the various modes of a given solid are equal to the same value γ, yielding the scaling law ω ~ *V^−^*^γ^, in which the frequency spectrum uniformly expands as the crystal contracts upon compression [24,47]. In molecular crystals like SBF, the mode Grüneisen parameters, apart from the mode frequency anharmonicity, express the force (bond stiffness) hierarchy. Although the Grüneisen approximation still works well for the intermolecular modes, it fails completely for the intramolecular modes, and a rough γ_i_ ~ $\omega_{i0}^{-2}$ dependence is observed, where ω_i0_ is the frequency of the *i*th mode at zero pressure [23,24]. As the bulk modulus for the SBF crystals is not available in the literature, and in order to compare its various binding forces, we can use the experimentally obtained logarithmic pressure coefficients of the frequencies (frequency normalized pressure slopes) Γ_i_ = ∂(lnω_i_)/∂*P*= (1/ω_i0_)·∂ω_i_/∂*P* of all the observed Raman peaks, which are proportional to the γ_i_ parameters (γ_i_ = *B*_0_Γ_i_).

**Figure 6:**
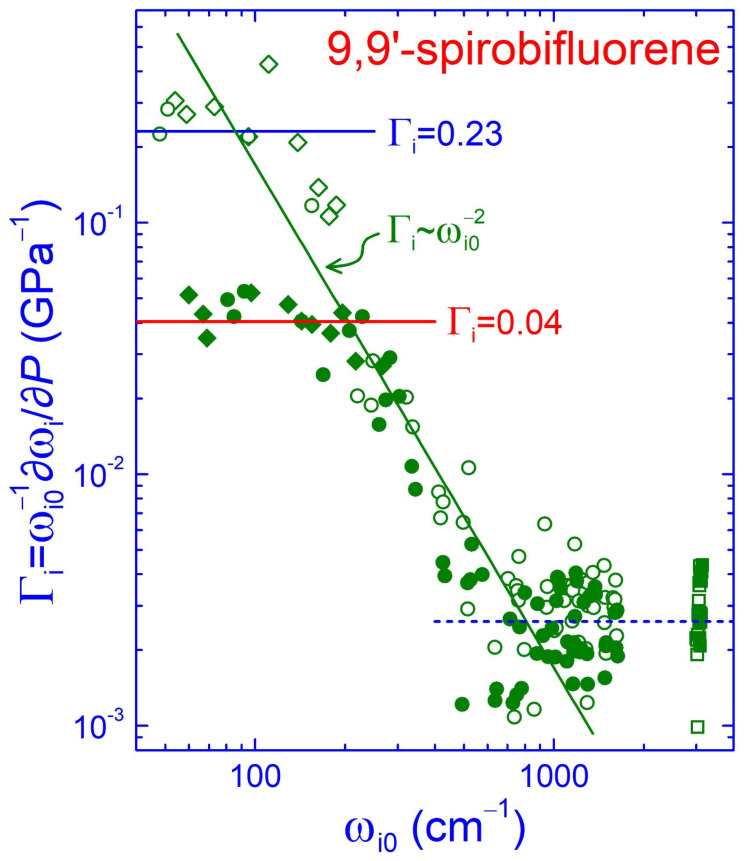
Frequency-normalized pressure slopes Γ_i_ = (1/ω_i0_)·∂ω_i_/∂*P* of the Raman peaks of SBF as a function of their frequency ω_i0_ at 0.0 GPa (open symbols—Γ_i_ values of the Raman peaks for *P* < 1.3 GPa) or 4.2 GPa (solid symbols—Γ_i_ values of the Raman peaks for *P* > 4.2 GPa). Rhombi, circles and squares refer to the external, internal and C–H stretching vibrations, respectively. The solid lines through the data correspond to Γ_i_ = constant (external modes) and Γ_i_ ~ $\omega_{i0}^{-2}$ (internal modes), while the dashed line corresponds to Γ_i_ = constant for the internal modes with ω_i0_ > 1000 cm^−1^.

The normalized pressure slopes Γ_i_ of all the intermolecular and intramolecular Raman peaks of SBF below 1.3 GPa (low-pressure phase—open symbols) or above 4.2 GPa (high-pressure phase—solid symbols) as a function of their frequency ω_i0_ at 0.0 or 4.2 GPa, respectively, are summarized in Figure 6. As can be inferred from this figure, the Γ_i_ parameters spanned more than two orders of magnitude in the low-pressure phase of SBF, reflecting the coexistence of the weak van der Waals intermolecular interactions and the strong covalent intramolecular bonding. The parameters were almost constant for the intermolecular modes (rhombi), with an averaged value of ~0.23 (blue horizontal line), whereas they roughly followed Γ_i_ ~ $\omega_{i0}^{-2}$ dependence (olive line) for the low- and intermediate-frequency intramolecular modes (circles). On the other hand, the Γ_i_ parameters were again nearly constant for the higher-frequency intramolecular modes (ω > 1000 cm^−1^), including the C–H stretching vibrations (squares). This behavior reflected the similar force constants involved in these vibrations.

From the averaged value of the Γ_i_ parameters for the intermolecular modes, we could obtain a rough estimation for the bulk modulus of SBF in the low-pressure phase, assuming that the corresponding Grüneisen parameter equaled 2 [23,24,25]. This estimation yielded *B*_0_ ~ 9 GPa (~2/0.23 GPa), which was of the same order of magnitude—though 50% larger—as that experimentally obtained for the parent fluorene crystals in the low-pressure phase, *B*_0_ ~ 6 GPa, with a pressure derivative of $B_{0}^{\prime }=$ 7.5–8 [46,48]. In the high-pressure phase, the corresponding XRD data showed that fluorene crystals became stiffer with *B*_0_ = 11.3 GPa and $B_{0}^{\prime }=$ 5.4 [46]. In the present case of SBF, its intense stiffening at 4.2 GPa, where the second phase transition or the completion of the structural phase transition at ~1.3 GPa occurred, was associated with the considerable strengthening of the intermolecular interactions. This was clearly revealed by the strong—almost by 6 times—decrease in the Γ_i_ parameters for the intermolecular and low-frequency intramolecular modes (solid symbols in Figure 6). These parameters at 4.2 GPa become more comparable to those of the intermediate- and high-frequency intramolecular modes, reflecting a more homogenized state of the studied system in terms of the strength of the binding forces.

## 3. Materials and Methods

**Figure A1:**
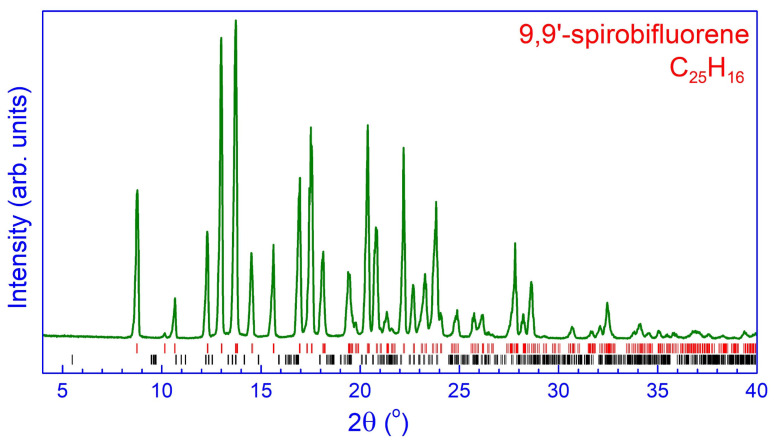
Powder X-ray diffraction profiles (λ = 1.5418 Å) of the studied crystalline SBF sample at ambient conditions. The red (upper) ticks mark the expected reflection positions for the main monoclinic SBF polymorph reported by Schenk [21], whereas the black (lower) ticks mark those for the metastable SBF polymorph reported by Douthwaite et al. [22].

The studied SBF (C_25_H_16_) crystals (purity > 98%) were purchased from Tokyo Chemical Industry Co., Ltd. (TCI, Tokyo, Japan). The as-received sample was structurally characterized by means of powder X-ray diffraction (XRD) using a Brücker D8 Advance diffractometer (Billerica, MA, USA) equipped with a Cu X-ray tube (Cu Kα radiation, λ = 1.5418 Å). The XRD profiles at ambient conditions were collected at 2θ from 3° to 40° with an increment of 0.01° and a 0.2 s scan time. The corresponding data are illustrated in Figure A1 (Appendix A) along with the reflection positions expected for the main monoclinic SBF polymorph reported by Schenk (CSD Entry: BIPHME, Deposition Number: 1111373) and those for the rather metastable one by Douthwaite et al. (CSD entry: BIPHME01, deposition number: 273066) [21,22,42]. From these, it was apparent that the studied material crystallized in the monoclinic structure reported by Schenk [21].

Raman spectra were acquired in the backscattering geometry using a micro-Raman LabRAM HR spectrometer (HORIBA, Kyoto, Japan) equipped with a Peltier-cooled charge-coupled detector (CCD). The 632.8 nm line of a He-Ne laser was used for excitation, focused on the sample by means of a 100× objective (focusing spot diameter: ~1 μm) for ambient pressure or a 50× objective (focusing spot diameter: ~2 μm) for the high-pressure measurements at a power lower than 0.4 or 2 mW, respectively, to avoid any laser heating-induced effects. The elastically scattered light from the sample was eliminated by means of an appropriate edge filter, while the inelastically scattered light was dispersed by a 600 g/mm grating. The combination of this grating with an entrance pinhole of 80 μm yielded a spectral width of ~3.5 cm^−1^. Prior to each measurement, the frequency axis in the Raman spectrum was calibrated by the corresponding spectral lines of a Ne lamp.

The high-pressure Raman experiments were conducted in a Mao-Bell-type diamond anvil cell (DAC) using a ~80 μm thick drilled (~120 μm hole diameter) stainless steel gasket [49]. Daphne 7474 oil, which is a non-polar medium and, hence, was expected to affect to a lesser extent the molecular crystals of SBF and their pressure response compared to polar fluids, was used as a pressure transmitting medium (PTM) [50]. This PTM solidified at 3.7 GPa at room temperature and exhibited very good hydrostatic behavior up to ~4.1 GPa since the standard deviation of pressure remained lower than 0.013 GPa. At higher pressures, the standard deviation increased quasi-linearly, reaching the value of 0.25 GPa for *P* = 8 GPa [51]. The pressure in the sample chamber inside the DAC was calibrated by means of the ruby fluorescence (R_1_ line) method [52,53].

## 4. Conclusions

In conclusion, we reported our detailed high-pressure Raman study of crystalline 9,9′-spirobifluorene (SBF) up to 8 GPa, using a diamond anvil cell for pressure generation and Daphne 7474 oil as a pressure transmitting medium. The abrupt changes observed for pressures higher than 1.3 GPa for the Raman peaks attributed to both external and internal vibrational modes and the pressure evolution of their frequencies signaled the occurrence of a pressure-induced phase transition of the studied material, characterized by structural and molecular conformation alterations. This phase transition, presumably of first-order in nature, showed a little hysteretic behavior and the initial phase was recovered at ~0.7 GPa upon pressure release. For pressures higher than 4.2 GPa, the linear pressure coefficients of the intermolecular and some low- and intermediate-frequency intramolecular modes reversibly decreased, indicating the occurrence of a second structural transition or the completion of the one initiated at ~1.3 GPa. From the low-pressure ω vs. *P* data for the intermolecular modes, we estimated a value of ~9 GPa for the bulk modulus of the low-pressure phase of crystalline SBF. In the high-pressure phase and at 4.2 GPa, the considerable strengthening of the intermolecular interactions significantly narrowed the initially broad range of the force constants in the SBF crystal.

## Data Availability

All data supporting the reported results in this paper can be obtained from the authors.

## Appendix A

**Table A1:** Mode symmetry in the *D*_2*d*_ point group, approximate mode description and calculated (ω_calc_, cm^−1^) and experimental (ω_exp_, cm^−1^) frequencies of the vibrational modes of 9,9′-spirobifluorene, as obtained from the literature [43]. The frequencies (ω_i0_ at 0.0 GPa, ω_j0_ at 1.1 GPa or at the lowest pressure they appear and ω_k0_ at 4.4 GPa or at the lowest pressure they appear in cm^−1^) extracted from the least-squares fits and the corresponding ω vs. *P* data of the present work, along with their linear (*b*_1_, cm^−1^GPa^−1^) and quadratic pressure coefficients (*b*_2_, cm^−1^GPa^−2^), are also presented. The corresponding data for the well-resolved Raman peaks are shown in bold. The symmetry, approximate mode description and ω_calc_ from [43] for the Raman peaks tentatively assigned in this work are shown in italics.

Peak	Sym.*	Approx. Mode Description *	ω_calc_ *	ω_exp_ **	*P* < 1.3 GPa			1.1 < *P* < 4.1 GPa			*P* > 4.2 GPa	
					ω_i0_	*b*_1_	*b*_2_	ω_j0_	*b*_1_	*b*_2_	ω_k0_	*b*_1_
ω_1_								**50**	**4.5**		**60**	**3.1**
ω_2_											69	2.4
ω_3_								**54**	**6.8**		**67**	**2.9**
ω_4_	B_1_	ring tor	47	48	**48**	**10.8**		**56**	**12.6**	**−0.95**	81	4.0
ω_5_								75	4.9		85	3.6
ω_6_	*E*	*ring tor and defm*	*46*		51	14.4		**63**	**12.6**	**−0.77**	**92**	**4.9**
ω_7_		*external*			54	16.5						
ω_8_		*external*			59	15.9		**72**	**13.1**	**−1.03**	97	5.1
ω_9_		*external*			73	21.1		95	23.3	−2.53	129	6.1
ω_10_								110	20.4	−1.64		
ω_11_	*E*	*ring tor and defm or external*	*92*		**95**	**20.9**		**106**	**22.1**	**−2.06**	**143**	**5.8**
ω_12_											155	6.1
ω_13_								121	27.5	−2.38	169	4.2
ω_14_								139	25.5	−2.50		
ω_15_		*external*			**111**	**47.4**	**−11.60**	163	8.6		**179**	**6.5**
ω_16_								155	13.9	−0.15	196	8.6
ω_17_		*external*			139	29.0						
ω_18_	B_1_	ring tor	156	156	155	18.1		172	15.7	−0.59	207	7.7
ω_19_		*external*			**163**	**22.5**		**177**	**24.8**	**−2.20**	217	6.1
ω_20_		*external*			177	18.8						
ω_21_		*external*			187	22.0		213	14.0		267	7.2
ω_22_	*B_2_*	*ring defm*	*216*		220	4.5		**217**	**−7.8**	**1.95**	**229**	**9.7**
ω_23_	*E*	*ring tor, ip (CH + CCC) bend*	*240*		245	4.6		**246**	**4.3**		**260**	**4.1**
ω_24_								259	11.3		274	5.4
ω_25_	*E*	*ring tor, ip (CH + CCC) bend*	*240*		248	7.0		260	9.8	−0.62	283	8.2
ω_26_								273	21.5	−2.17		
ω_27_								285	6.2			6.2
ω_28_	B_1_	ring tor	319	320	**321**	**6.5**		323	3.6			3.6
ω_29_	A_1_	ip CH and CCC bend	331	336	**337**	**5.2**		**335**	**3.0**			**3.0**
ω_30_	B_2_	ring defm	407	412	412	3.5						
ω_31_	E	ring tor	424	419	418	2.8		420	1.9			1.9
ω_32_	A_1_	ring defm	420	424	426	3.3		427	1.7			1.7
ω_33_								487	3.5	−0.25	494	0.6
ω_34_	*B_1_*	*oop CH and CCC bend*	*501*		498	3.2		502	6.3		515	1.9
ω_35_	E	ring tor, ip (CH + CCC) bend	513	514	515	1.5		516	2.9		526	2.0
ω_36_	*E*	*ring tor, ip (CH + CCC) bend*	*513*		519	5.5		520	3.7		532	2.8
ω_37_								569	2.3			2.3
ω_38_	E	ring tor, ip (CH + CCC) bend	644	636	635	1.3		633	0.8			0.8
ω_39_								640	1.9	−0.10	644	0.9
ω_40_	A_1_	ip CH and CCC bend	705	704	**703**	**2** **.7**		**708**	**2.7**	**−0.12**	**715**	**1.9**
ω_41_	E	oop CH and CCC bend	735	727	728	0.2		728	0.9			0.9
ω_42_	*E*	*oop CH and CCC bend*	*735*		738	0.8						
ω_43_	E	oop CH bend	752	750	750	2.7		750	1.0			1.0
ω_44_	*E*	*oop CH bend*	*752*		756	2.6						
ω_45_	A_1_	ip CH and CCC bend	766	760	763	2.4		764	1.9			1.9
ω_46_	*B_1_*	*oop CH bend*	*762*		765	3.6		769	7.8	−0.79	781	1.1
ω_47_	B_1_	oop CH and CCC bend	800	796	796	1.6		795	0.3	0.25	800	2.7
ω_48_	*E*	*oop CH bend*	*864*		861	1.0		854	1.1		858	0.2
ω_49_								869	5.3	−0.44	879	1.7
ω_50_								875	2.7			2.7
ω_51_								915	2.1			2.1
ω_52_	*B_1_*	*oop CH and CCC bend*	*943*		932	5.9						
ω_53_	E	oop CH and CCC bend	947	946	948	2.8		**951**	**1.8**			**1.8**
ω_54_	B_2_	ip CH and CCC bend	948	948	951	3.4						
ω_55_	*E*	*oop CH and CCC bend*	*947*		959	0.4						
ω_56_								980	2.4			2.4
ω_57_	B_1_, E	oop CH and CCC bend, ip CH and CCC bend	984, 1005	1004	**1005**	**2.4**		1008	1.9			1.9
ω_58_											1020	3.2
ω_59_	A_1_, B_2_	ip CH and CCC bend	1020	1020, 1021	1020	2.5		**1026**	**4.0**			**4.0**
ω_60_	E	ip CH and CCC bend	1035	1031	1029	4.0		1041	3.7			3.7
ω_61_	A_1_	ip CH and CCC bend	1083	1088	1084	3.4						
ω_62_	B_2_	ip CH and CCC bend	1105	1104	1105	0.7		1102	2.0			2.0
ω_63_	*E*	*ip CH and CCC bend*	*1103*		1110	4.0		1105	2.4			2.4
ω_64_								1111	2.4			2.4
ω_65_	A_1_	ip CH and CCC bend	1148	1152	**1152**	**3.0**		**1155**	**1.7**			**1.7**
ω_66_	*E*	*ip CH and CCC bend*	*1155*		1157	4.2		1158	2.3			2.3
ω_67_	E	ip CH and CCC bend	1155	1164	1160	4.0		1163	2.5			2.5
ω_68_								1170	3.2			3.2
ω_69_								1171	4.8			4.8
ω_70_	A_1_, E	ip CH and CCC bend	1178, 1179	1176	1175	6.2		1182	4.5			4.5
ω_71_	B_2_	C(sp^3^)—C str, ip CH and CCC bend	1206	1208	1210	2.6						
ω_72_	*B_2_*	*ip CH and CCC bend*	*1222*		1214	3.8		1215	2.4			2.4
ω_73_	A_1_	ring str, ip CH bend	1221	1224	**1226**	**4.6**		**1226**	**2.5**			**2.5**
ω_74_					1232	4.7						
ω_75_								1249	3.9			3.9
ω_76_	E	ip CH and CCC bend	1288	1282	1282	2.6		1286	2.5			2.5
ω_77_	B_2_	ip CH and CCC bend	1299	1295	**1294**	**1.6**		**1294**	**1.9**			**1.9**
ω_78_	A_1_	ip CH and CCC bend	1298	1297	**1297**	**4.8**						
ω_79_	*E*	*ip CH and CCC bend*	*1305*		1301	3.9		1304	4.3			4.3
ω_80_	B_2_	ip CH and CCC bend	1352	1351	1352	5.5		1360	4.9			4.9
ω_81_	*A_1_*	*ip CH and CCC bend*	*1354*	1352	1359	4.0		1368	4.6			4.6
ω_82_								1477	2.3			2.3
ω_83_	E	ip CH and CCC bend	1473	1474	1476	6.4						
ω_84_	A_1_	ip CH and CCC bend	1471	1480	**1480**	**3.8**		**1485**	**3.1**			**3.1**
ω_85_	*B_2_*	*ip CH and CCC bend*	*1474*	1480	**1485**	**4.8**		1488	3.2			3.2
ω_86_					1498	2.9						
ω_87_	A_1_, B_2_	ip CH and CCC bend	1573, 1572	1580, 1581	1581	5.1		1587	4.5			4.5
ω_88_					1591	4.7						
ω_89_								1609	3.3			3.3
ω_90_	*E*	*ip CH and CCC bend*	*1594*		1601	3.3						
ω_91_	E	ip CH and CCC bend	1594	1604	1605	5.1		1609	4.6			4.6
ω_92_	A_1_	ip CH and CCC bend	1596	1608	**1610**	**6.1**		**1614**	**4.7**			**4.7**
ω_93_	B_2_	ip CH and CCC bend	1591	1628	1624	3.7		1630	3.1			3.1
ω_94_		*2nd order*			3004	6.7						
ω_95_		*2nd order*			3012	6.6		3019	8.7			8.7
ω_96_	*E*	*CH str*	*3047*		3024	5.8						
ω_97_	*E*	*CH str*	*3047*		**3033**	**3.0**		3036	7.8			7.8
ω_98_	*B_2_*	*CH str*	*3048*		3040	7.0						
ω_99_	*A_1_*	*CH str*	*3048*		**3047**	**8.4**		3060	6.9			6.9
ω_100_	*A_1_*	*CH str*	*3058*		3060	9.6		**3068**	**6.4**			**6.4**
ω_101_											3100	8.0
ω_102_	*A_1_*	*CH str*	*3068*		**3065**	**11.3**		**3081**	**8.7**			**8.7**
ω_103_	*E*	*CH str*	*3067*		3075	11.1		**3081**	**11.7**			**11.7**
ω_104_	*A_1_*	*totally sym CH str*	*3080*		3081	13.3		**3092**	**8.6**			**8.6**
ω_105_								**3105**	**12.8**			**12.8**
ω_106_	*B_2_*	*CH str*	*3080*		3088	12.3		3099	13.7			13.7
ω_107_								3118	11.7			11.7
ω_108_		*2nd order*			3159	9.4		3171	6.4			6.4
ω_109_		*2nd order*			3196	10.1		3222	7.2			7.2
ω_110_		*2nd order*			3209	13.8		3218	9.9			9.9

* Symmetries, approximate mode descriptions and ω_calc_ values are those extracted from the DFT calculations (4–31 G basis set) by Boo et al. [43]. Approximate mode description: tor, torsion; defm, deformation; bend, bending; str, stretching; sci, scissoring; twi, twisting; roc, rocking; ip, in-plane; oop, out-of-plane. ** ω_exp_ values are taken from the Raman spectroscopic measurements of SBF crystals and saturated solution in CCl_4_ by Boo et al. [43].
